# Cyanoacrylate tissue adhesive

**DOI:** 10.4103/0301-4738.64129

**Published:** 2010

**Authors:** Viroj Wiwanitkit

**Affiliations:** Wiwanitkit House, Bangkhae, Bangkok-101 60, Thailand

Dear Editor,

I read the recent publication on cyanoacrylate tissue adhesive by Romero *et al*. with great interest.[1] Romero *et al*, conclude that the polymerization reaction might also be an important factor in the antibacterial properties of liquid ethyl-cyanoacrylate (EC) and N-butyl-cyanoacrylate (BC).[1] Indeed, the use of cyanoacrylate tissue adhesive as fibrin glue is accepted for its efficacy in medicine. The bactericidal activity of EC and BC reported in the paper by Romero *et al*,[1] is of interest. The bactericidal activity of this kind of medical product has been mentioned for a very long time.[2] The polymerization reaction can play a role as raised by Romero *et al*.[1] Based on the concept of nanomedicine, the polymerization reaction is a kind of exothermic reaction, constructing bonds between molecules,[3,4] and this can be the source of energy that result in bactericidal outcome.
